# Parenteral Fish-Oil Containing Lipid Emulsions Limit Initial Lipopolysaccharide-Induced Host Immune Responses in Preterm Pigs

**DOI:** 10.3390/nu13010205

**Published:** 2021-01-12

**Authors:** William Yakah, David Ramiro-Cortijo, Pratibha Singh, Joanne Brown, Barbara Stoll, Madhulika Kulkarni, Berthe C. Oosterloo, Doug Burrin, Krishna Rao Maddipati, Raina N. Fichorova, Steven D. Freedman, Camilia R. Martin

**Affiliations:** 1 Beth Israel Deaconess Medical Center, Department of Neonatology, Harvard Medical School, 330 Brookline Avenue, Boston, MA 02115, USA; wyakah@bidmc.harvard.edu; 2 Beth Israel Deaconess Medical Center, Division of Gastroenterology, Harvard Medical School, 330 Brookline Avenue, Boston, MA 02115, USA; dramiro@bidmc.harvard.edu (D.R.-C.); psingh6@bidmc.harvard.edu (P.S.); jbrown17@bidmc.harvard.edu (J.B.); sfreedma@bidmc.harvard.edu (S.D.F.); 3 Children’s Nutrition Research Center, United States Department of Agriculture-Agricultural Research Service, Department of Pediatrics, Baylor College of Medicine, 1100 Bates Street, Houston, TX 77030, USA; bstoll@bcm.edu (B.S.); neelkeoosterloo@gmail.com (B.C.O.); doug.burrin@usda.gov (D.B.); 4 Section Neonatology, Department of Pediatrics, Baylor College of Medicine, 1100 Bates Street, Houston, TX 77030, USA; mkulkarn@bcm.edu; 5 Lipidomics Core Facility, Department of Pathology, Wayne State University, 42 W Warren Avenue, Detroit, MI 48202, USA; maddipati@wayne.edu; 6 Gynecology and Reproductive Biology, Brigham and Women’s Hospital, Laboratory of Genital Tract Biology, Department of Obstetrics, Harvard Medical School, 75 Francis Street, Boston, MA 02115, USA; rfichorova@bwh.harvard.edu; 7 Beth Israel Deaconess Medical Center, Division of Translational Research, Harvard Medical School, 330 Brookline Avenue, Boston, MA 02115, USA

**Keywords:** fatty acids, arachidonic acid, lipid metabolism, eicosanoids, oxylipins, metabolomics, sphingomyelin, acute inflammation, preterm pig

## Abstract

Multicomponent lipid emulsions are available for critical care of preterm infants. We sought to determine the impact of different lipid emulsions on early priming of the host and its response to an acute stimulus. Pigs delivered 7d preterm (*n* = 59) were randomized to receive different lipid emulsions for 11 days: 100% soybean oil (SO), mixed oil emulsion (SO, medium chain olive oil and fish oil) including 15% fish oil (MO15), or 100% fish oil (FO100). On day 11, pigs received an 8-h continuous intravenous infusion of either lipopolysaccharide (LPS—lyophilized *Escherichia coli*) or saline. Plasma was collected for fatty acid, oxylipin, metabolomic, and cytokine analyses. At day 11, plasma omega-3 fatty acid levels in the FO100 groups showed the highest increase in eicosapentaenoic acid, EPA (0.1 ± 0.0 to 9.7 ± 1.9, *p* < 0.001), docosahexaenoic acid, DHA (day 0 = 2.5 ± 0.7 to 13.6 ± 2.9, *p* < 0.001), EPA and DHA-derived oxylipins, and sphingomyelin metabolites. In the SO group, levels of cytokine IL1β increased at the first hour of LPS infusion (296.6 ± 308 pg/mL) but was undetectable in MO15, FO100, or in the animals receiving saline instead of LPS. Pigs in the SO group showed a significant increase in arachidonic acid (AA)-derived prostaglandins and thromboxanes in the first hour (*p* < 0.05). No significant changes in oxylipins were observed with either fish-oil containing group during LPS infusion. Host priming with soybean oil in the early postnatal period preserves a higher AA:DHA ratio and the ability to acutely respond to an external stimulus. In contrast, fish-oil containing lipid emulsions increase DHA, exacerbate a deficit in AA, and limit the initial LPS-induced inflammatory responses in preterm pigs.

## 1. Introduction

The third trimester of pregnancy is an essential phase of the growing human fetus, marked by an exponential increase in brain and lung maturation as well as an accumulation of adequate immunonutrients in adipose tissues [1,2]. The preterm infant is challenged with adapting from a controlled environment with biologically programmed nutritional supply to an external environment with high bacterial exposure and the requirement of exogenous nutritional support to meet its high metabolic needs [3]. Preterm birth results in early, postnatal deficits in long-chain polyunsaturated fatty acids (LCPUFAs), specifically docosahexaenoic acid (DHA) and arachidonic acid (AA) [4].

Lipid emulsions such as Intralipid^®^, a commonly used soybean-oil emulsion, contains essential fatty acid precursors such as linoleic acid (LA) but little to no AA and/or DHA [5]. Despite its benefits in providing a parenteral fat source, the use of Intralipid^®^ has been associated with increased pro-inflammatory markers [6] and parenteral nutrition associated cholestasis (PNAC) [7,8], when compared to the use of fish oil-containing emulsions containing omega-3 (*n*-3) LCPUFAs. Fish oil-containing emulsions such as Omegaven^®^ have been shown to reverse parenteral nutrition-associated cholestasis in infants [9,10,11]. Yet, the high EPA content in these lipid emulsions can result in decreased plasma AA levels [12]. Retrospective cohort analyses have shown that lower AA levels are associated with an increased risk of chronic lung disease, late-onset sepsis, and retinopathy of prematurity in preterm infants [4,13]. Moreover, low levels of AA may reduce the generation of AA derived proinflammatory eicosanoids during the acute phase of the inflammatory response.

The inflammatory response to harmful stimuli is an important physiological defense mechanism necessary to maintain homeostasis [14,15]. This process is marked by two significant events: (1) an initial release of pro-inflammatory prostaglandins that drive neutrophil activation to engulf pathogens, and (2) a subsequent pro-resolving response involving lipid mediator class-switching and clearance of apoptotic cells by macrophages to resolve inflammation [16,17]. While growing evidence supports that eicosapentaenoic acid (EPA) and DHA-enriched lipid supplementation is effective in suppressing inflammation [6], it is unclear how alterations in LCPUFA profiles affect processes involved in systemic inflammation such as sepsis, especially in preterm infants with an immature immune system.

In this study, we examined the effect of nutritional priming with different lipid emulsions on lipopolysaccharide (LPS)-induced acute inflammatory responses using a preterm pig model [18,19]. We hypothesized that parenteral administration of different lipid emulsions containing a gradient of increasing n-3 LCPUFAs results in a dominant balance of *n*-3 relative to *n*-6 fatty acid levels, and *n*-3 dominance attenuates LPS-induced acute inflammatory responses through changes in plasma oxylipin, cytokine, and metabolomic mediators in preterm pigs. These results will expand our understanding of the role of LCPUFAs in disease risk in the preterm population and begin to inform optimal fatty acid delivery strategies to support the unique metabolic requirements of the preterm infant.

## 2. Materials and Methods

### 2.1. Animal Care and Surgery

The study was conducted in accordance with the Declaration of Helsinki, and the protocol was approved by the Ethics Committee of Baylor College of Medicine (Houston, TX, USA) meeting the requirements of the U.S. Public Health Service Policy on Humane Care and Use of Laboratory Animals (Ref. D16-00475).

Pigs from nine sows were used in this study. Timed-pregnant sows were obtained from a commercial swine farm, housed at the animal facility of the Children’s Nutrition Research Center with food and water provided ad libitum. Preterm pigs were delivered by cesarean section at 108 days of gestation (115 days term) and placed in acrylic incubators housed at 32 °C as described previously [18,19]. Pigs delivered 7 days preterm (about 93% gestation) possess clinical signs and organ maturity levels consistent with moderate prematurity (32 weeks’ gestation and greater) and represent an established model to study nutrition in the preterm infant [20,21,22]. After delivery, pigs underwent surgery for placement of jugular catheters for parenteral nutrition (PN) administration and PN was initiated following line placement. During the first 24 h, maternal plasma (16 mL/kg in 3 doses) was administered IV for passive immunological protection. Postoperatively, pigs were monitored continuously over the 10 d study period, including rectal temperatures and vital signs (oxygen saturation and heart rate) twice daily. Nutrition rates were adjusted according to body weights taken every other day. Growth rates (g/kg/d) were calculated by:

(final weight, g,−birth weight, g)/(average (final weight, kg; birth weight, kg))/11 d

### 2.2. Nutrition Protocol and Study Design

**Figure 1:**
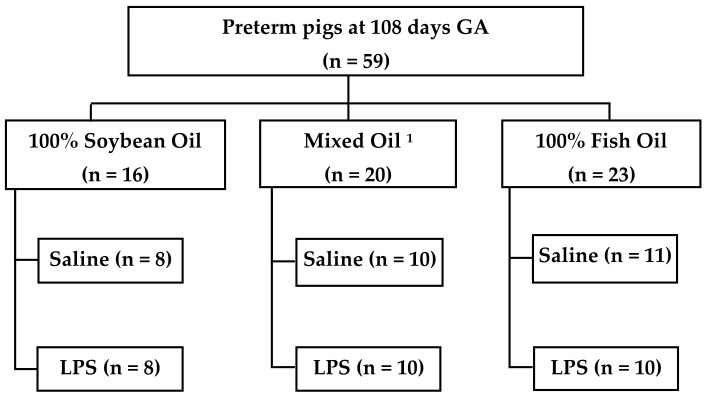
Experimental study design. Pigs were delivered 7 days preterm and received total parenteral nutrition for 11 days. On day 11, pigs received either LPS (lyophilized *E. Coli*) or saline. ^1^Mixed oil contains: 30% soybean oil, 30% medium chain triglycerides, 25% olive oil, 15% fish oil. GA, gestational age; LPS, lipopolysaccharide.

Following surgery on day 0, pigs received PN at 5.5 mL/kg·h (132.5 mL/kg·d or 50% of total intake). At this rate, PN provided (per kg/d): 506 kJ energy, 12.5 g dextrose, 6.5 g amino acids, 5.0 g fat. Infusion rates were gradually increased to 100% within the following 7 days such that nutrient intakes met the daily requirement for neonatal pigs. Fat in the form of lipid emulsions was administered at 10g/kg·d as in previously PN-fed pig studies [23,24]. Pigs were randomized on day 0 to receive one of three lipid emulsions (all from Fresenius Kabi, Bad Homburg, Germany) (Figure 1): (a) SO, 100% soybean oil (Intralipid^®^ 20%, *n* = 16); (b) MO15, mixed oil containing 30% SO, 30% medium chain triglycerides, 25% olive oil, 15% fish oil (SMOFlipid^®^ 20%, *n* = 20); or (c) FO100, 100% fish oil (Omegaven^®^ 10%, *n* = 23).

These lipid emulsions were not added to the PN-bags but infused separately via syringe pump before joining the jugular catheter using a Y-connector. Because Omegaven^®^ was provided as a 10% lipid emulsion, both, Intralipid^®^ and SMOFlipid^®^ (20% lipid emulsions) were diluted 1:1 with sterile water in order to administer equal volumes of PN to all pigs. Due to the required dilution, it was necessary to concentrate the remainder of the PN solution to maintain the daily intake of dextrose, amino acids, electrolytes, vitamins and trace minerals used in previous studies [18,25]. Overall, the PN infusion rate at full intake was 11 mL/kg/h.

### 2.3. LPS Infusion Protocol

On day 11, pigs in all three groups were randomly assigned to (±LPS) treatment (Figure 1). All pigs in the LPS groups received an 8 h continuous infusion (10 µg/kg·h) of *Escherichia coli* endotoxin (lyophilized *E. coli* Serotype 0111-B4, Sigma Chemical, St. Louis, MO, USA) while the control groups received an equal volume of sterile saline solution (0.9% sodium chloride). The LPS (5 mg/mL in 0.9% saline) or 0.9% saline only was added to the respective lipid emulsion and thus continuously infused into the intravenous catheter via Y-connection along with total PN solution. Vital signs including, heart rate, SpO_2_, and rectal temperature were measured each hour during the LPS or saline infusion. Jugular blood samples were collected into EDTA tubes on days 0, 11, and hourly during the 8 h continuous LPS or saline infusion, and then processed to separate plasma and red blood cells. Both fractions were frozen in separate tubes and stored at −80 °C. All pigs were euthanized at the end of the 8 h protocol.

### 2.4. Fatty Acid Analysis

Fatty acids were isolated and methylated using a modified Folch method as previously described [4,26]. Fatty acid in plasma samples were quantified by gas chromatography–mass spectroscopy (GC-MS) with a Supelcowax-10 column (Sigma-Aldrich, Saint Luis, MO, USA). Peak identification was based upon comparison of both retention time and mass spectra of the unknown peak to that of known standards. Fatty acid methylated ester (FAME) mass was determined by comparing areas of unknown FAMEs to that of a fixed concentration of 17:0 internal standard. Response factors were determined for each individual FAME to correct for GC-MS total ion chromatogram discrepancies in quantification. These factors were determined through the use of a GLC reference standard which contained known masses of FAMEs ranging from 14–24 °C. The response ratio of each FAME is corrected to a fixed amount ratio for each FAME relative to 17:0. Individual FA is expressed as a percent of the total FA mass (mol%).

### 2.5. Cytokine Measurements

Inflammatory cytokines were quantified hourly in plasma samples during the LPS challenge. Six cytokines including C-reactive protein (CRP), interleukin (IL)1b, IL6, IL8, IL10, and tumor necrosis factor alpha (TNFα) were analyzed using an electrochemiluminescent multiplex detection platform (MesoScale Discovery, Gaithersburg, MD, USA) with precision and accuracy previously validated by comparison to international standards and traditional ELISA [27]. Replicates of a quality control pool were tested in each assay with an inter-assay coefficient of variation (100 × SD/average) ranging from 11% to 33% (20.7 ± 7.9%). For the purposes of statistical analyses, cytokine values below the lowest limit of detection (LLOD) were replaced with ½ LLOD specified by the assay, as previously established [28]. Analyte values were reported as pg/mL with LLOD for IL1b = 3.41, IL6 = 5.96, IL8 = 1.77, IL10 = 0.93, TNFα = 0.4 and CRP = 0.64.

### 2.6. Oxylipin Analysis

Plasma oxylipin levels at birth, day 11 at hours 0, 1, 4, and 7 were analyzed using Liquid chromatography-mass spectrometry (LC-MS) by the Lipidomics Core Facility, Wayne State University School of Medicine (Detroit, MI, USA) as previously described [29]. Briefly, 100 μL of plasma was supplemented with 5ng each of a mix internal standard consisting of prostaglandin E_1_-d4, Resolvin D2-d5, leukotriene B_4_-d4, 15-HETE-d8, and 14(15)-EpETrE-d11 (Cayman Chemical, Ann Arbor, MI, USA), and lipid metabolites isolated by solid phase extraction on a Strata X column (30 mg sorbent, 1 mL; Phenomenex, Torrance, CA, USA). The extracted samples were evaporated, reconstituted in methanol-water (1:1), and oxylipins were resolved by HPLC (Luna, C18(2); 2.1 × 150 mm, 3 µm; Phenomenex). The eluted oxylipins were analyzed by QTRAP55500 mass analyzer (AB Sciex, Framingham, MA, USA) using electrospray ionization source operated in the negative-ionization mode via multiple-reaction monitoring (MRM) method using transitions that were optimized for selectivity and sensitivity, as described earlier. The data were collected with Analyst 1.6.3 software (AB Sciex, Framingham, MA, USA), and the MRM transition chromatograms were quantitated by MultiQuant software (AB Sciex, Framingham, MA, USA) using quantification against deuterated internal standards. For the purposes of statistical analyses, oxylipin values below limit of detection were replaced by half of the minimum detected level (i.e., 0.001 nM).

Plasma metabolomic analyses were analyzed by Metabolon, Inc. (Durham, NC, USA). Each sample was accessioned into the Metabolon laboratory information management system (LIMS) and assigned a unique identifier. All samples were prepared using the automated MicroLab STAR^®^ system from Hamilton Company. The LC-MS portion of the platform was based on a Waters ACQUITY ultra-performance liquid chromatography (UPLC) and a Thermo Scientific Q-Exactive high resolution/accurate mass spectrometer interfaced with a heated electrospray ionization (HESI-II) source and Orbitrap mass analyzer operated at 35,000 mass resolutions. Raw data were extracted, peak identified and quality control process using Metabolon’s hardware and software. Compounds were identified by comparison to library entries of purified standard or recurrent unknown entities. Peaks were quantified using area-under-the-curve.

### 2.7. Statistical Analysis

All reported measures were evaluated for normality. Quantitative measures were summarized as median ± interquartile range (IQR). For survival curve analysis, log-rank test was performed. For comparisons in pig vital signs, plasma fatty acid, and metabolomic analyses across different lipid groups, two-way ANOVA for main effects of lipid group (SO, MO15, FO100) and Day (day 0, day 11). For each analysis, a generalized linear modeling following rank-normal transformation of data was used to compare unbalanced, repeated measures during the course of experiment. Differences in oxylipin levels were determined using median fold change during the priming stage (day 11 compared to day 0) and LPS challenge (LPS at hour 1, 4, and 7 compared to Day 11). Significance of differences were determined by two-way ANOVA using rank-normalized data with lipid group (SO, MO15, FO100) and Day (Day 0, Day 11) as main effects.

Data analyses were performed using R software (version 3.5.2, R Core Team 2018a) within RStudio (Version 1.1.453, RStudio, Inc., Vienna, Austria) using the tidyverse [30] phyloseq [31], and multcomp [32] packages. Heatmaps were generated using pheatmap [33] package. We defined biological significance as oxylipins or metabolites with median fold changes ≥ |2.0|. *p*-values were adjusted for false discovery rate (FDR) using Benjamini–Hochberg approach, and statistical significance was considered at *p* < 0.05.

## 3. Results

### 3.1. Pig Cohort Characteristics

A total of 59 pigs (16, 20, and 23 in SO, MO15, and FO100 lipid groups, respectively) were included in this study. Cohort characteristics among lipid groups showed no significant difference in birth weight, weight at day 11, or growth rates across the three lipid groups (Table 1).

**Figure 2:**
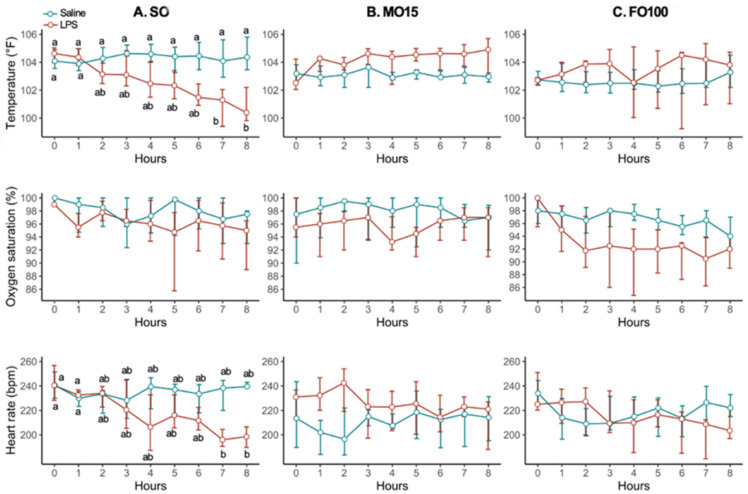
Vital sign changes in pigs during LPS challenge. Hourly recordings in temperature, oxygen saturation, and heart rate by lipid group, SO (**A**, *n* = 16), MO (**B**, *n* = 20), and FO100 (**C**, *n* = 23) lipid groups. Data was presented as line plot showing median and IQR. Groups without common letters are significantly different (*p* < 0.05). FO100, 100 percent fish oil; LPS, lipopolysaccharide; MO15, mixed oil with 15% fish oil; SO, 100% soybean oil.

Vital signs (temperature, oxygen saturation, and heart rate) recorded during LPS infusion stratified by lipid and treatment groups are shown in Figure 2. Within the SO group, pigs receiving LPS infusion by hour 8 had significantly decreased median temperature compared to hour 0 (104.6 ± 1.1 vs. 100.4 ± 2.4, *p* = 0.02) and decreased heart rate at hour 8 compared to hour 0 (240.5 ± 28.5 vs. 198.5 ± 16.8, *p* = 0.03). No significant difference in vital signs were recorded within the MO15 and FO100 lipid groups.

### 3.2. Fatty Acid Changes

**Figure 3:**
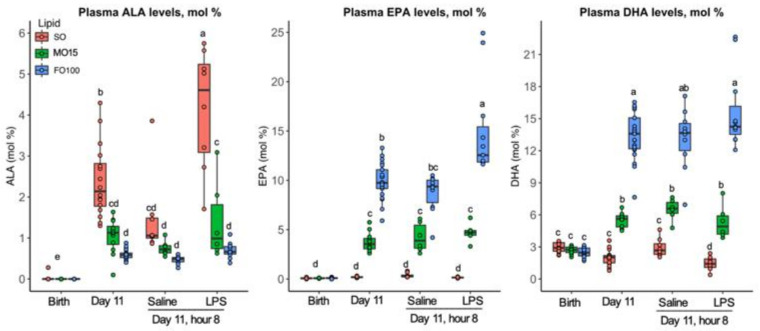
Specific plasma *n*-3 fatty acid profiles across postnatal age as a function of lipid group. Concentrations of fatty acids were determined by GC-MS and are presented as mol% in the form of boxplots with dots. Data show median and IQR. At day 11, pigs in each lipid group received either LPS or saline, and fatty acid levels were measured at 8 h at the end of the continuous infusion. Labeled points without a common letter represent a statistically significant difference of *p* < 0.05. ALA, α-linolenic acid; DHA, docosahexaenoic acid; EPA, eicosapentaenoic acid; FO100, 100 percent fish oil; MO15, mixed oil with 15% fish oil; SO, 100% soybean oil.

**Figure 4:**
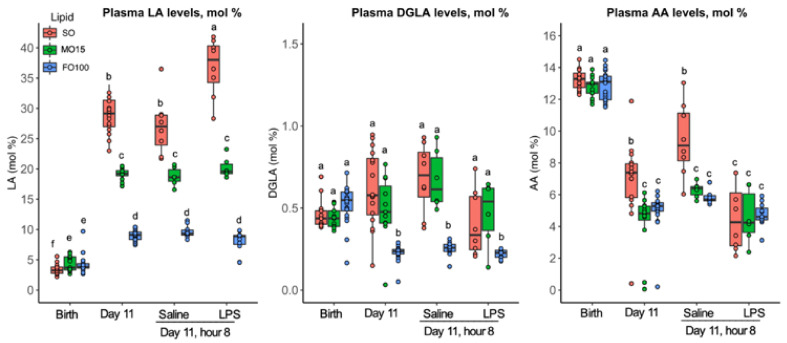
Specific plasma *n*-6 fatty acid profiles across postnatal age as a function of lipid group. Concentrations of fatty acids were determined by GC-MS and are presented as mol% in the form of boxplots with dots. Data show median and IQR. At day 11, pigs in each lipid group received either LPS or Saline, and fatty acid levels were measured at 8 h at the end of the continuous infusion. Labeled points without a common letter represent a statistically significant difference of *p <* 0.05. AA, arachidonic acid; DGLA, dihomo-γ linolenic acid; FO100, 100 percent fish oil; LA, linoleic acid; MO15, mixed oil with 15% fish oil; SO, 100% soybean oil.

Changes in specific *n*-3 (ALA, EPA, DHA) and *n*-6 (LA, DGLA, AA) fatty acids in plasma over the 11-day protocol are shown in Figure 3 and Figure 4, respectively. Birth levels of ALA, EPA, and DHA did not differ between lipid groups. During the priming stage (from birth to day 11, prior to LPS infusion), the SO lipid group showed significantly higher ALA at day 11 compared to birth levels (0.0 ± 0.0 to 2.1 ± 1.0, *p* < 0.001), but no significant difference in EPA and DHA during the priming stage (Figure 3, Supplementary Material Table S1). In the fish-oil lipid groups, the FO100 group during their priming stage showed the highest increase in EPA (0.1 ± 0.0 to 9.7 ± 1.9, *p* < 0.001) and DHA (2.5 ± 0.7 to 13.6 ± 2.9, *p* < 0.001). When fatty acid levels were measured 8 h after continuous LPS infusion at day 11, the SO group showed a significant increase in ALA (Saline = 1.1 ± 0.5; LPS = 4.6 ± 2.1, *p* < 0.001), while the FO100 group showed a significant increase in EPA (Saline = 9.4 ± 2.3; LPS = 12.5 ± 3.6, *p* < 0.001) but not DHA (Saline = 13.7 ± 2.6; LPS = 14.2 ± 2.7, *p* = 0.72). No significant differences in plasma levels of EPA or DHA were observed in SO and MO15 groups after LPS infusion.

Similarly, plasma changes in specific *n*-6 (LA, DGLA, AA) fatty acids are shown in Figure 4. Birth levels of *n*-6 FAs did not differ by lipid groups, but significantly increased in a gradient manner during the priming stage (Figure 4, Supplementary Material Table S1). At day 11, levels of LA in the SO group were the highest, followed by MO15 and FO100 lipid groups. Compared to saline, the level of LA in the fish oil groups did not change after LPS infusion but increased in the SO group (saline = 27.0 ± 5.0; LPS = 38.0 ± 6.0, *p* = 0.001). AA levels significantly decreased in all three-lipid groups at day 11 compared to birth levels, and the LPS infusion did not result any significant change in AA levels in all lipid groups.

**Figure 5:**
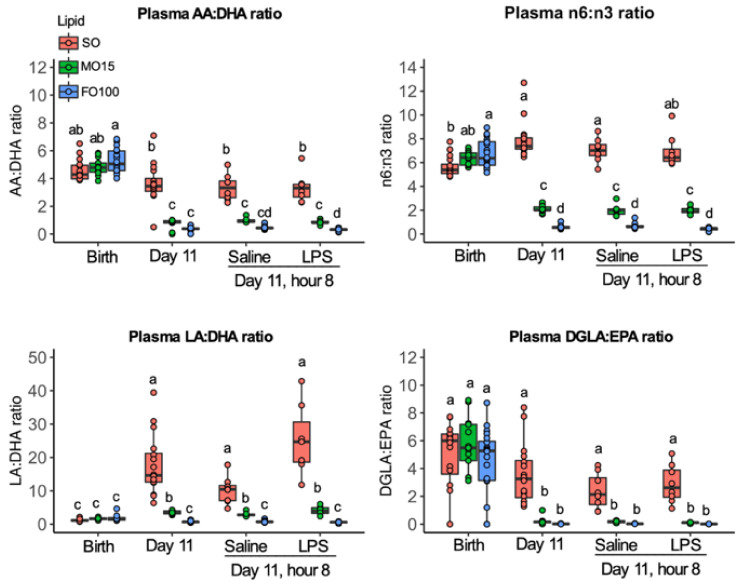
Plasma fatty acid ratios across postnatal age as a function of lipid group. Concentrations of fatty acids were determined by GC-MS and are presented as mol% in the form of boxplots with dots. Data show median and IQR. At day 11, pigs in each lipid group received either LPS or Saline, and fatty acid levels were measured at 8 h at the end of the continuous infusion. Labeled points without a common letter represent a statistically significant difference of *p <* 0.05. AA, arachidonic acid; DGLA, dihomo-γ linolenic acid; EPA, eicosapentaenoic acid; FO100, 100 percent fish oil; LA, linoleic acid; MO15, mixed oil with 15% fish oil; *n*-3, omega-3, *n*-6, omega-6; SO, 100% soybean oil.

The ratio of AA:DHA did not differ by lipid groups at birth, but significantly decreased at day 11 in MO15 (4.8 ± 0.5 to 0.9 ± 0.1, *p* < 0.001) and FO100 (5.1 ± 1.4 to 0.4 ± 0.1, *p* < 0.001) (Figure 5, Supplementary Material Table S1). In contrast, in the SO group, the ratio of AA:DHA at birth (4.3 ± 1.0), day 11 (3.4 ± 0.9), or 8 h after LPS administration (3.3 ± 0.9) did not significantly change.

### 3.3. Plasma Oxylipin Changes

**Figure 6:**
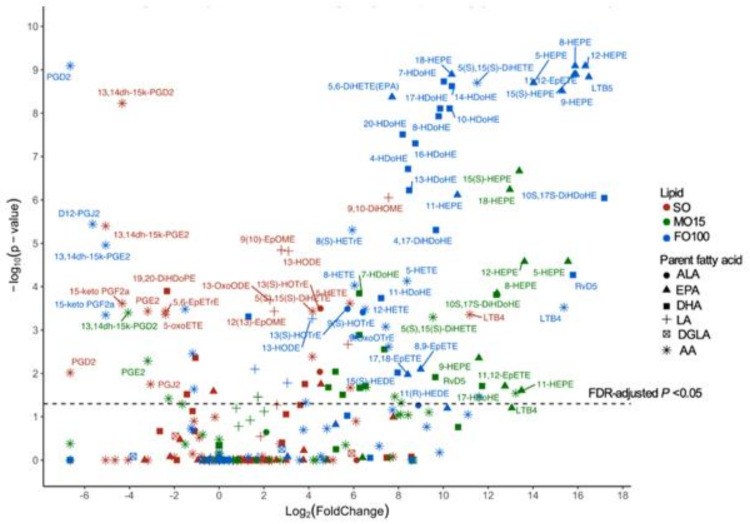
Changes in plasma oxylipins at day 11 compared to day zero (priming stage) by lipid group, SO (*n* = 10), MO15 (*n* = 9), and FO100 (*n* = 10). Scatter plot shows the median fold change of oxylipins within each lipid group indicated by colors and parent fatty acids of the oxylipins indicated by shapes. AA, arachidonic acid; ALA, α-linolenic acid; DGLA, dihomo-γ linolenic acid; DHA, docosahexaenoic acid; EPA, eicosapentaenoic acid; FO100, 100 percent fish oil; LA, linoleic acid; MO15, mixed oil with 15% fish oil; SO, 100% soybean oil.

Changes in plasma oxylipins were measured at two time points during the priming stage (day 0 and day 11) and at three time points during LPS challenge at day 11 (hours 1, 4, and 7). Oxylipin levels during the priming stage (day 11 compared to day 0) within each lipid group are shown in Figure 6. Compared to birth levels in the fish-oil groups, many EPA-derived hydroxy-eicosapentaenoic acids (HEPEs) and DHA-derived hydroxy FAs (HDoHEs) were significantly increased in the fish oil groups. In contrast, AA-derived prostaglandin derivatives (PGE2, PGD2 and PGF2α) were significantly decreased, while levels of leukotriene B4 (LTB4) increased in all three-lipid groups. Additionally, the SO group showed a significant increase in LA-derived (e.g., EpOMEs, DiHOMEs) oxylipins during the priming stage, which were not seen in fish-oil groups.

**Figure 7:**
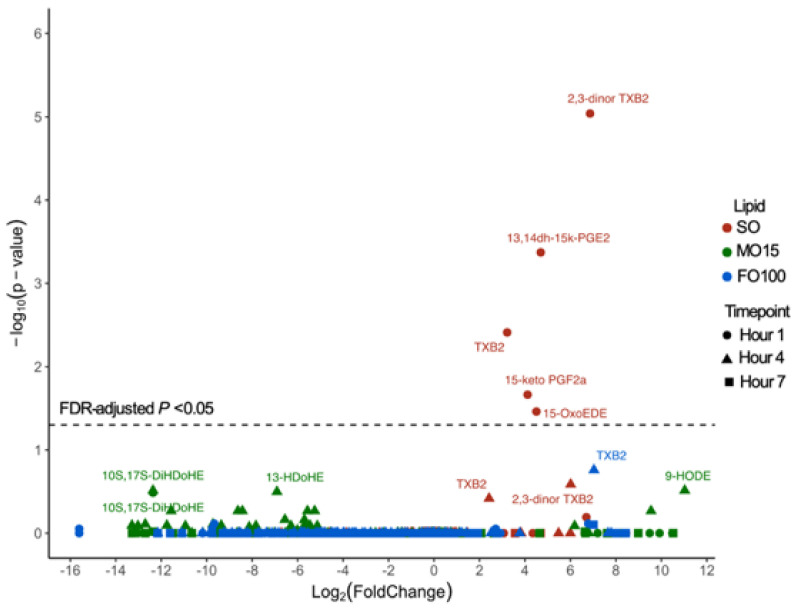
Changes in plasma oxylipins during LPS infusion. Scatter plot shows the median fold change of oxylipins at different hours of infusion (shape) compared to baseline on day 11 within each lipid group, SO (red, *n* = 10), MO15 (green, *n* = 9), and FO100 (blue, *n* = 10). AA, arachidonic acid; ALA, α-linolenic acid; DGLA, dihomo-γ linolenic acid; DHA, docosahexaenoic acid; EPA, eicosapentaenoic acid; FO100, 100 percent fish oil; LPS, lipopolysaccharide; LA, linoleic acid; MO15, mixed oil with 15% fish oil; SO, 100% soybean oil.

Within each lipid group, oxylipin levels during different time points in pigs who received LPS were compared to that of saline at day 11 and expressed as log2 fold changes in Figure 7. Interestingly, only the SO group showed a response to LPS infusion, significantly increasing AA-derived thromboxane B2 (TXB2), 2,3-dinor TXB2, prostaglandins 13,14, dihidro-15keto-PGE2, 15-keto-PGF2α, and eicosadienoic acid-derived 15-Oxo-EDE at the first hour. Other than a modestly significant increase in TXB2 at hours 4 and 7 in FO100 group, no other significant change in oxylipin response was seen in the fish-oil groups during LPS challenge.

### 3.4. Cytokine and Metabolomic Changes

**Figure 8:**
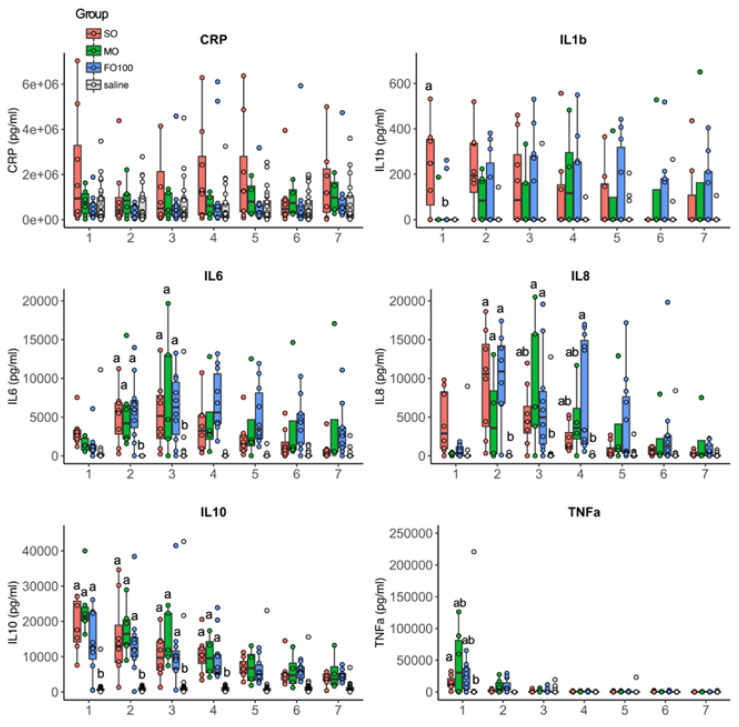
Plasma levels of C-reactive protein (CRP), interleukin (IL)-1b, IL-6, IL-8, IL-10 and tumor necrosis factor (TNF)-α by lipid group, (SO (*n* = 8), MO15 (*n* = 6), and FO100 (*n* = 11), recorded hourly after the introduction of lipopolysaccharide on day 11. Data show median and IQR. Labeled points without a common letter represent a statistically significant difference of *p <* 0.05. FO100, 100 percent fish oil; MO15, mixed oil with 15% fish oil; SO, 100% soybean oil.

At day 11, plasma levels of cytokines CRP, IL6, IL8, IL10, and TNFα were quantified during 8 h LPS infusion protocol (Figure 8). At the first hour of LPS infusion, levels of IL1b were detected only in the SO group (296.6 ± 308.0 pg/mL) but below the limit of detection in MO15, FO100, or saline groups. Levels of IL6, IL8, and IL10 and TNFα were highest in the first couple of hours and decreased over the period of LPS infusion. These levels did not significantly differ between lipid groups.

**Figure 9:**
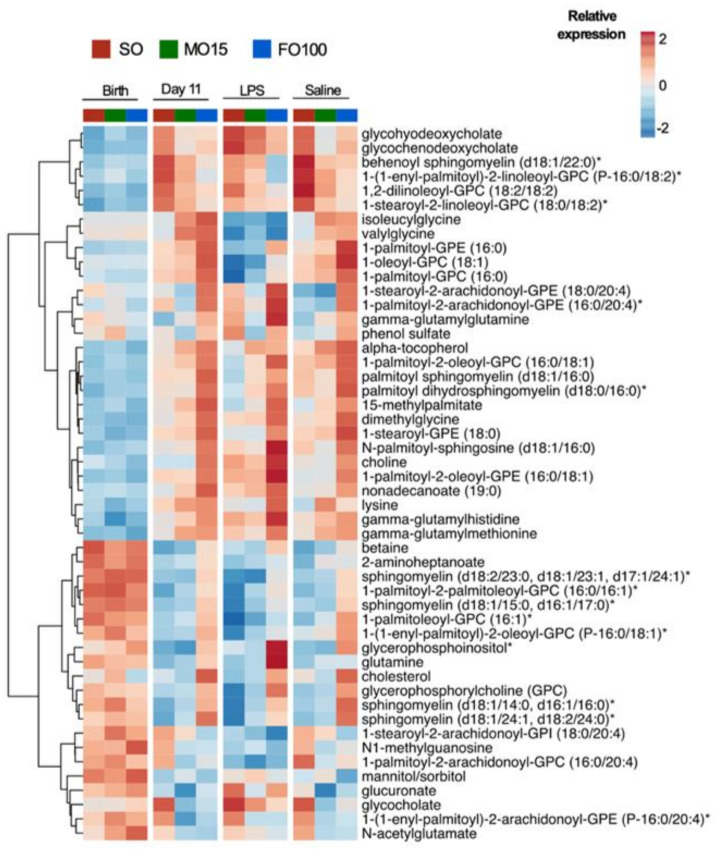
Changes in plasma lipid metabolites at birth, day 11, and at the end of an 8 h continuous infusion of saline or LPS on day 11 by lipid group, SO (*n* = 16), MO15 (*n* = 13), and FO100 (*n* = 19). The heatmap shows the median relative expression of the top 50 significantly different lipid metabolites by 2-way ANOVA (lipid × day) per lipid groups. FO100, 100 percent fish oil; LPS, lipopolysaccharide; MO15, mixed oil with 15% fish oil; SO, 100% soybean oil.

Metabolomic profiles of 313 unique plasma metabolites were measured at birth and day 11 before and after LPS treatment, and the top 50 significant metabolites are shown as a heatmap in Figure 9. At birth, the relative expression of metabolites was similar in all three-lipid groups. However, at day 11, metabolites differed by lipid groups, with increased choline and its downstream glycerophospholipids and sphingomyelin metabolites in FO100 compared to MO15 and SO lipid groups. LPS treatment showed similar metabolomic profiles compared to saline treatment within lipid groups.

## 4. Discussion

The objective of this study was to investigate the effect of parenteral LCPUFA priming on the systemic, acute inflammatory response in a preterm pig model. The preterm TPN-fed pig provides a clinically relevant model of preterm infants to study the physiological effects of different commercially available lipid emulsions. Our results show that changes in systemic fatty acid levels reflect the composition of LCPUFAs in the different lipid emulsions inducing lipid specific oxylipin and metabolic changes. Priming with fish oil-containing lipid emulsions reduced the initial host inflammatory responses to an LPS-induced trigger in preterm pigs.

Changes in fatty acid levels observed in blood plasma reflected levels in the respective lipid emulsions received parenterally. Increased levels of EPA and DHA in fish-oil groups have also been observed in serum of preterm infants receiving fish-oil containing parenteral lipid emulsions [34]. However, our results also show that birth levels of AA are not maintained in any of the three lipid emulsion groups. Although the plasma level of LA, an *n*-6 fatty acid precursor, increased in SO group at day 11, the plasma levels of AA, a downstream product of LA metabolism did not increase at day 11 compared to that of birth level. Previous studies in preterm infants have shown that endogenous synthesis of AA from upstream precursors is limited and decreases with advancing development [35,36]. Furthermore, measurements of desaturase activity in both term and preterm infants have shown that downstream elongation and desaturation in the *n*-6 pathway is most limited by conversion of LA to DGLA (20:3n6) in the pathway of AA synthesis [37,38]. These results suggest that additional nutritional supplementation of AA may be necessary to maintain birth levels and prevent a postnatal deficit. Maintaining birth levels of AA is critical to neonatal development, as a postnatal decrease in AA has been associated with increased risk of neonatal morbidities such as late-onset sepsis and retinopathy of prematurity [4,13]. We have previously shown in a pig model that providing enteral lipid emulsions with an AA:DHA ratio > 1 maintained birth levels of AA and improved intestinal development in preterm pigs [39]. Together, these data suggest that it is not only individual fatty acids such as AA and DHA, but the ratio of these fatty acids are crucial for neonatal development.

The conversion of LCPUFAs to their bioactive oxygenated products (i.e., oxylipins) depend on the relative composition of both *n*-3 and *n*-6 fatty acids, since both groups compete for the same enzymes [40,41,42]. As such, an *n*-3 dominant state such as the fish-oil groups, reflected by increased plasma EPA and DHA levels, drives the production of more EPA and DHA-derived oxylipins during the priming stage. The high *n*-3 derived oxylipins predispose the host to an anti-inflammatory, pro-resolving state by reducing polymorphonuclear neutrophil (PMN) infiltration, platelet aggregation, and expression of proinflammatory cytokines [43,44]. In contrast, the decreased fatty acid levels of AA during the priming stage, which corresponded to decreased AA-derived oxylipins in all lipid groups, potentially due to DHA outcompeting AA for incorporation into membrane phospholipids, or the reduction of AA from elevated EPA levels [12,45,46], reduced AA derived oxylipins synthesis. Biosynthesis of oxylipins (prostaglandins, leukotrienes, thromboxanes) through AA metabolism modulates the inflammatory response in the host by producing both pro- and anti-inflammatory mediators [47,48].

In an immature host such as the preterm infant, the ratio between *n*-3 and *n*-6 lipid mediators may influence the host’s immune response to acute inflammation. The n6:n3 ratio present at birth was maintained with in the SO group but was significantly decreased in the MO15 and FO100 groups in a dose-dependent manner relative to the content of fish oil. Our results show that the highly dominant *n*-3 fatty acid and the respective oxylipin levels blunt the host’s IL1β cytokine response to LPS-induced systemic inflammation in the fish-oil groups. Additionally, only the pigs in the SO group that received LPS showed a significant increase in prostaglandins 13,14dh-15k-PGE_2_, 15-keto PGF_2α_ and thromboxanes TXB_2_, 2,3-dinor TXB_2_ in the first hour of LPS infusion. The presence of an acute systemic response in the SO group was supported clinically by the observation of decreased body temperature early during the LPS infusion. These results are supported by previous findings in animal models that also reported an increase in TBX_2_, PGE_2_, and PGF_2α_ in macrophages and neutrophils a few hours after LPS challenge [49,50,51]. This initial inflammatory response increases recruitment of PMNs and stimulates the production of pro-inflammatory cytokines by macrophages as a necessary first-line defense against infection [52]. This initial response is mediated by the cyclooxygenase-2 (COX-2) enzyme product PGE_2_, which is the most abundant eicosanoid at the onset of infection in humans [53] and has both pro- and anti-inflammatory properties. PGE_2_ induces a pro-inflammatory response through influx of neutrophils and macrophages [51,54], and later, mediates the resolution phase of inflammation through lipid mediator class switching [55,56] and increased anti-inflammatory cytokine, IL10 [57]. It is worth noting that while LPS infusion continued for 8-h, prostaglandin oxylipins in the SO group was only significantly elevated at the first hour, possibly due to an insufficient amount of AA, which serves as a precursor to PGE_2_ synthesis, to sustain continuous PGE_2_ synthesis. Alternatively, this transient rise in PGE_2_ may be a normal physiologic response to an infection to limit the inflammatory responses generated. Together, these findings support the essentiality of AA in modulating inflammation, while also demonstrating that *n*-3 dominant lipid emulsions reduce cytokine and pro-inflammatory responses during the initiation phase of the inflammatory response to a LPS challenge. Our study was limited to evaluating the acute inflammatory response. It is possible that with later, chronic inflammation, the need for *n*-3 fatty acids, such as DHA and EPA, are critical to activate the second, pre-resolution, phase of inflammation [43].

Metabolomic profiles also showed increased plasma choline and sphingomyelin metabolites in the *n*-3-dominant FO100 lipid group at day 11 compared to birth levels. Sphingomyelin metabolites have been shown to modulate inflammatory signaling by binding to proinflammatory mediators and endotoxins such as LPS in lipid rafts and is a possible marker of sepsis [58]. In animal studies, decreased sphingomyelin content in macrophages correspond with reduced TLR4, TNFα and NF-κB activation [59,60] and attenuated LPS-induced macrophage inflammation [61]. Additionally, sphingomyelin is hydrolyzed by sphingomyelinase (SMase) to ceramide, a bioactive lipid with structural similarity to bacterial LPS and modulates immune response through similar proinflammatory mechanisms [62,63]. In an *n*-3 dominant lipid group, increased DHA has been shown to reduce SMase activity, thus decreasing ceramide synthesis and a concomitant decrease in inflammatory response [64]. However, this decrease in SMase activity may be reversed by introduction of LPS endotoxin, which has been shown to increase SMase and subsequently, ceramide synthesis and inflammation [65]. Thus, the counteracting effects of DHA on SMase activity to reduce ceramide synthesis, as evidenced by accumulation of sphingomyelin metabolites, may also account for the initial decreased host’s response to acute stimuli in the fish oil groups.

Though oxylipin production can be linked directly to the overall fatty acid content in the lipid emulsions and resultant changes in systemic fatty acid levels, the metabolomic changes quantified reflect the impact of all compositional elements packaged together as a lipid emulsion product. For example, factors, other than fatty acids may drive these metabolomic responses such as phospholipid, including choline content derived from the phospholipids, and vitamin E content, two factors known to differ between 10% and 20% lipid emulsions and in emulsions with higher concentrations of long chain polyunsaturated fatty acids [66]. Our study adds to growing evidence that nutrient priming drives host immune and metabolic responses [67], thus it is critical to examine lipid emulsions from several angles, from their overall systemic effects as one product and by its specific elemental components. Disentangling the compositional drivers from complex matrices such as lipid emulsions would require additional examinations and may be helpful in determining optimal lipid emulsions compositions for the preterm infant.

## 5. Conclusions

In summary, our results show that the composition of parenteral lipid emulsions significantly influences systemic fatty acid profiles in preterm pigs. Additionally, priming of an *n*-3-dominant lipid emulsion decreases AA and AA-derived oxylipins, primarily PGE2 metabolites and sphingomyelin metabolism, thereby limiting the initial LPS-induced host responses.

## Figures and Tables

**Table 1:** Piglet cohort characteristics by lipid groups.

Lipid	Treatment	*n*	Male	Birth Weight (g)	Final Weight (g)	Growth Velocity (g/kg/d)	Mortality
100% Soybean Oil (SO)	**Total**	**16**	**68.8%**	**1273 (362)**	**2521 (605)**	**98.4 (9.73**	**1 (6.3%)**
	Saline	8		1380 (382)	2569 (603)	96.3 (11.1)	1 (12.5%)
	LPS	8		1243 (356)	2515 (614)	98.8 (7.77)	0 (0.0%)
Mixed Oil (MO15)	**Total**	**20**	**60.0%**	**1199 (274)**	**2387 (474)**	**101.0 (12.8)**	**2 (10.0%)**
	Saline	10		1189 (240)	2387 (364)	105.0 (9.55)	1 (10.0%)
	LPS	10		1211 (298)	2378 (523)	97.0 (16.7)	1 (10.0%)
100% Fish Oil (FO100)	**Total**	**23**	**52.2%**	**1249 (177)**	**2438 (373)**	**98.7 (19.0)**	**3 (13.0%)**
	Saline	12		1228 (150)	2438 (450)	97.9 (16.7)	0 (0.0%)
	LPS	11		1265 (142)	2444 (313)	100.5 (17.4)	3 (25.0%)

Data show median and interquartile range (IQR) in quantitative variables and sample size and relative frequency (%) in quantitative variables. Final weight was at the 8th hour of day 11, or the last weight if the piglet died before finishing the study. LPS, lipopolysaccharide.

## Data Availability

The data presented in this study are available on request from the corresponding author. The availability of the data is restricted to investigators based in academic institutions.

## Supplementary Materials

The following are available online at https://www.mdpi.com/2072-6643/13/1/205/s1, Table S1: Plasma levels of selected fatty acids in the *n*-3 and *n*-6 pathways expressed as mol% and selected fatty acid ratios.

## Abbreviations

AA—Arachidonic Acid; ALA—alpha-Linolenic Acid; CRP—C-reactive Protein; DGLA—Dihomo-gamma-Linolenic Acid; DHA—Docosahexaenoic Acid; EPA—Eicosapentanoic Acid; FAME—Fatty Acid Methylated Ester; FO—Fish Oil; GA—Gestational Age; GC-MS—Gas Chromatography–Mass Spectroscopy; HDoHEs—DHA-derived hydroxy Fatty Acids; HEPEs—Hydroxy-Eicosapentaenoic Acids; IL—interleukin; LA—Linoleic Acid; LCPUFAs—Long-Chain Polyunsaturated Fatty Acids; LPS—Lipopolysaccharide; LT—Leukotriene; MO—Mixed Oil; MRM—Multiple Reaction Monitoring; PG—Prostaglandin; PMN—Polymorphonuclear Neutrophil; PN—Parenteral Nutrition; PNAC—Parenteral Nutrition Associated Cholestasis; SO—Soybean Oil; TLR—Toll-like Receptor; TNFα—Tumor Necrosis Factor alpha; TX—Thromboxane; UPLC—Ultra-Performance Liquid Chromatography.
