# Supplementary material for: Parenteral Fish-Oil Containing Lipid Emulsions Limit Initial Lipopolysaccharide-Induced Host Immune Responses in Preterm Pigs
Source: Nutrients. 2021 Jan 12;13(1):205. doi: 10.3390/nu13010205 (PMC7828127; doi:10.3390/nu13010205)
Supplement: Supplementary file 1 [file nutrients-13-00205-s001.pdf]

nutrients

MDPI

Table S1: Plasma levels of selected fatty acids in the n-3 and n-6 pathways expressed as mol% and selected fatty acid ratios.

Fatty Acid	Day	Hour	Group	100% Soybean Oil	Mixed Oil	100% Fish Oil
18:3n-3 ALA	0			0.0 (0.0-0.0)^{e}	0.0 (0.0-0.0)^{e}	0.0 (0.0-0.0)^{e}
	11	0		2.1 (1.8-2.8)^{b}	1.1 (0.8-1.3)^{cd}	0.6 (0.5-0.6)^{d}
	11	8	Saline	1.1 (1.0-1.5)^{cd}	0.7 (0.6-0.8)^{d}	0.5 (0.4-0.5)^{d}
	11	8	LPS	4.6 (3.1-5.2)^{a}	1.0 (0.7-1.8)^{c}	0.7 (0.6-0.8)^{d}
20:5n-3 EPA	0			0.1 (0.1-0.1)^{s}	0.1 (0.1-0.1)^{d}	0.1 (0.1-0.1)^{d}
	11	0		0.2 (0.2-0.2)^{d}	3.6 (3.1-4.1)^{c}	9.74 (9.2-11.1)^{b}
	11	8	Saline	0.3 (0.2-0.4)^{d}	3.9 (3.1-5.5)^{c}	9.4 (7.7-10.0)^{bc}
	11	8	LPS	0.2 (0.1-0.2)^{d}	4.7 (4.4-4.9)^{c}	12.5 (11.8-15.4)^{a}
22:6n-3 DHA	0			2.9 (2.6-3.4)^{c}	2.7 (2.5-3.0)^{c}	2.5 (2.2-2.9)^{c}
	11	0		2.0 (1.5-2.2)^{c}	5.6 (4.9-5.9)^{b}	13.6 (12.2-15.1)^{a}
	11	8	Saline	2.7 (2.3-3.3)^{c}	6.6 (6.2-7.2)^{b}	13.7 (12.0-14.6)^{ab}
	11	8	LPS	1.4 (1.1-1.9)^{d}	4.9 (4.2-5.9)^{b}	14.2 (13.5-16.2)^{a}
18:2n-6 LA	0			3.3 (2.8-3.8)^{f}	3.7 (3.3-5.5)^{e}	3.8 (3.6-4.3)^{e}
	11	0		29.2 (27.0-1.8)^{b}	19.2 (18.9-19.7)^{c}	9.1 (8.3-9.6)^{d}
	11	8	Saline	27.0 (23.9-28.9)^{b}	18.6 (18.0-19.9)^{c}	9.3 (9.1-9.9)^{d}
	11	8	LPS	38.0 (34.3-40.3)^{a}	19.6 (19.2-20.8)^{c}	8.9 (7.5-9.0)^{d}
20:3n-6 DGLA	0			0.4 (0.4-0.5)^{a}	0.4 (0.4-0.5)^{a}	0.5 (0.5-0.6)^{a}
	11	0		0.6 (0.5-0.8)^{a}	0.5 (0.4-0.7)^{a}	0.2 (0.2-0.2)^{b}
	11	8	Saline	0.7 (0.6-0.8)^{a}	0.6 (0.5-0.8)^{a}	0.3 (0.2-0.3)^{b}
	11	8	LPS	0.3 (0.2-0.6)^{a}	0.5 (0.4-0.6)^{a}	0.2 (0.2-0.2)^{b}
20:4n-6 AA	0			13.3 (12.7-13.6)^{a}	13.0 (12.4-13.1)^{a}	13.1 (12.0-13.5)^{a}
	11	0		7.4 (5.8-7.9)^{b}	4.8 (4.4-5.3)^{c}	5.3 (5.0-5.5)^{c}
	11	8	Saline	9.1 (8.1-11.1)^{b}	6.4 (6.0-6.5)^{c}	5.7 (5.6-5.9)^{c}
	11	8	LPS	4.3 (2.8-6.1)^{c}	4.2 (3.6-6.1)^{c}	4.6 (4.4-5.2)^{c}
AA: DHA ratio	0			4.3 (4.0-5.0)^{ab}	4.8 (4.6-5.1)^{ab}	5.1 (4.6-6.0)^{a}
	11	0		3.4 (3.1-4.0)^{b}	0.9 (0.9-1.0)^{c}	0.4 (0.3-0.4)^{c}
	11	8	Saline	3.3 (2.6-3.8)^{b}	0.9 (0.8-1.0)^{c}	0.4 (0.4-0.5)^{c}
	11	8	LPS	3.3 (2.7-3.6)^{b}	0.8 (0.8-0.9)^{c}	0.3 (0.3-0.4)^{d}
n-6:n-3 ratio	0			5.4 (5.1-5.9)^{b}	6.4 (5.8-6.8)^{ab}	6.4 (5.8-7.8)^{a}
	11	0		7.4 (7.1-8.1)^{a}	2.0 (2.0-2.1)^{c}	0.6 (0.5-0.6)^{d}
	11	8	Saline	7.0 (6.6-7.5)^{a}	2.0 (1.7-2.1)^{c}	0.6 (0.6-0.7)^{d}
	11	8	LPS	6.4 (6.1-7.1)^{ab}	1.9 (1.8-2.1)^{c}	0.5 (0.4-0.5)^{d}
LA:DHA ratio	0			1.1 (1.0-1.3)^{d}	1.6 (1.4-1.9)^{d}	1.7 (1.2-2.0)^{d}

	11	0		14.6 (12.6-21.2)^{ab}	3.6 (3.1-3.9)^{c}	0.7 (0.5-0.7)^{d}
	11	8	Saline	10.4 (7.1-11.6)^{b}	2.8 (2.6-3.0)^{c}	0.7 (0.6-0.8)^{d}
	11	8	LPS	25.2 (18.9-37.5)^{a}	4.0 (3.3-5.0^{c}	0.6 (0.5-0.7)^{d}
DGLA:EPA ratio	0			6.0 (3.0-6.5)^{a}	5.5 (4.6-7.2)^{a}	5.3 (3.1-6.0)^{a}
	11	0		3.3 (1.9-4.6)^{a}	0.2 (0.1-0.2)^{b}	0.02 (0.01-0.03)^{b}
	11	8	Saline	2.1 (1.4-3.3)^{a}	0.2 (0.1-0.2)^{b}	0.03 (0.03-0.03)^{b}
	11	8	LPS	2.6 (1.9-3.9)^{a}	0.1 (0.1-0.1)^{b}	0.02 (0.01-0.02)^{b}

Data show median (Q1-Q3). Day 0 means day of birth. Medians without a common letter within each fatty acid group are statistically different p<0.05. AA, arachidonic acid; ALA, α-linolenic acid; DGLA, dihomo-γ linolenic acid; DHA, docosahexaenoic acid; EPA, eicosapentaenoic acid; FO100, 100 percent fish oil; LA, linoleic acid; LPS, lipopolysaccharide; MO15, mixed oil with 15% fish oil; n-3, omega-3; n-6, omega-6; SO, 100% soybean oil.
